# Neonatal near-miss audits: a systematic review and a call to action

**DOI:** 10.1186/s12887-023-04383-6

**Published:** 2023-11-17

**Authors:** P.B. Medeiros, C. Bailey, D. Pollock, H. Liley, A. Gordon, C. Andrews, V. Flenady

**Affiliations:** 1 Centre of Research Excellence in Stillbirth, Mater Research Institute, The University of Queensland, Brisbane, QLD Australia; 2 Sunshine Coast University Hospital, Sunshine Coast, QLD Australia; 3 JBI, School of Public Health, University of Adelaide, Adelaide, SA Australia; 4 Mater Research, Faculty of Medicine, The University of Queensland, Brisbane, QLD Australia; 5 University of Sydney, Sydney, NSW Australia

**Keywords:** Audit, Neonatal near miss, Perinatal morbidity, Perinatal mortality

## Abstract

**Background:**

Neonatal near-miss (NNM) can be considered as an end of a spectrum that includes stillbirths and neonatal deaths. Clinical audits of NNM might reduce perinatal adverse outcomes. The aim of this review is to evaluate the effectiveness of NNM audits for reducing perinatal mortality and morbidity and explore related contextual factors.

**Methods:**

PubMed, Embase, Scopus, CINAHL, LILACS and SciELO were searched in February/2023. Randomized and observational studies of NNM clinical audits were included without restrictions on setting, publication date or language. Primary outcomes: perinatal mortality, morbidity and NNM. Secondary outcomes: factors contributing to NNM and measures of quality of care. Study characteristics, methodological quality and outcome were extracted and assessed by two independent reviewers. Narrative synthesis was performed.

**Results:**

Of 3081 titles and abstracts screened, 36 articles had full-text review. Two studies identified, rated, and classified contributing care factors and generated recommendations to improve the quality of care. No study reported the primary outcomes for the review (change in perinatal mortality, morbidity and NNM rates resulting from an audit process), thus precluding meta-analysis. Three studies were multidisciplinary NNM audits and were assessed for additional contextual factors.

**Conclusion:**

There was little data available to determine the effectiveness of clinical audits of NNM. While trials randomised at patient level to test our research question would be difficult or unethical for both NNM and perinatal death audits, other strategies such as large, well-designed before-and-after studies within services or comparisons between services could contribute evidence. This review supports a Call to Action for NNM audits. Adoption of formal audit methodology, standardised NNM definitions, evaluation of parent’s engagement and measurement of the effectiveness of quality improvement cycles for improving outcomes are needed.

**Supplementary Information:**

The online version contains supplementary material available at 10.1186/s12887-023-04383-6.

## Background

Worldwide, more than 12,000 perinatal deaths (stillbirths and neonatal deaths) occur daily [1, 2]. There is a strong imperative to reduce perinatal mortality as many of these deaths are preventable [3, 4]. Structured multidisciplinary review of cases and identification of preventable factors is considered an important strategy to improve care and reduce adverse outcomes [3]. Perinatal mortality audit is described as “a systematic way of improving quality of care through collecting and analysing data, linking solutions and ensuring accountability for changes in care” (p.103) [5]. Evidence-based high-quality audit cycles can improve understanding of perinatal adverse outcomes and are acknowledged as the cornerstone of future prevention of perinatal deaths [6–8].

The term neonatal near-miss (NNM) commonly refers to a newborn who experienced severe, almost fatal complications of antenatal or intrapartum events, but survived [9]. It is therefore often perceived as one end of a spectrum that includes stillbirth and neonatal death. Originating in the aviation industry, the expression ‘‘near-miss’’ was later transposed to the health sector. Through the systematic study of near-miss accidents, centres that investigate and qualify airline services attempt to understand the chain of events leading to an accident and seek improvement. Transposing this principle, by identifying and auditing NNM, the database of cases for assessing maternal and perinatal care would be expanded [10]. There is a lack of international consensus on the criteria for NNM and there is potential for confusion with other classifications or syndromes such as sudden infant death syndrome (SIDS), Brugrada Syndrome and apparent life-threatening events (ALTE) [10–13]. In this study we use the term to apply to life-threatening events that originated in the antenatal or intrapartum period. The aim of identification of NNM events is to improve health care outcomes through systematic analysis of specific cases, preferably using multidisciplinary audit processes [14, 15].

It has been postulated that auditing NNM would increase opportunities for assessing and improving maternal and perinatal care and therefore might reduce morbidity and help prevent perinatal deaths [10, 13, 16]. Even if perinatal deaths are not reduced, NNM events are worth preventing for their own sake because serious illness around the time of birth can lead to lifelong adverse outcomes impacting health, wellbeing, lifespan and economic status of the child and their family [10, 17].

Some neonatal services already audit severe cases of neonatal morbidity. However, combined, multidisciplinary perinatal mortality and NNM audit has potential to draw wider conclusions about causal and avoidable factors than audits that separate the cases. Such an approach might be beneficial especially where perinatal mortality is already low [14, 18, 19]. A comparable method is currently used by Each Baby Counts in the United Kingdom, a quality improvement (audit) programme to reduce perinatal mortality and severe disability as a result of incidents occurring during term labour [20]. In this audit, each stillbirth, early neonatal death, and case of severe brain injury undergoes review by at least two independent clinical experts. Combining perinatal mortality and NNM audit might also improve opportunities to recognise whether there is a distribution drift, where improvements in mortality lead to more infants surviving with severe morbidity, as has been identified for maternal near-miss [21].

There is a growing body of evidence regarding identification and use of NNM cases by health facilities. Observational studies of NNM cases have described incidence, trend, associated factors, and generated meaningful insights such as possible prevention strategies [22–27]. Nevertheless, there is a dearth of evidence and to date it is not known what the best approach is for an effective NNM clinical audit and if the results of such programs have a positive and measurable impact on perinatal morbidity and mortality.

The primary aim of this systematic review was to determine whether for healthcare facilities, the use of NNM audits alters perinatal mortality and morbidity. Secondary aims were to determine whether NMM audits alter (1) identification of contributing care factors in NNM and (2) quality of pregnancy care. Additional goals were to garner information on NNM audits including feasibility, cost effectiveness and ability to generate targeted recommendations, as well as perceived facilitators and barriers to NNM audits.

## Methods

This review was reported according to the to the Preferred Reporting Items for Systematic Reviews and Meta-Analyses (PRISMA) guidelines. A comprehensive study protocol has been published elsewhere [28] and registered through PROSPERO (PROSPERO CRD42021224090).

### Information sources and search strategies

A search strategy was developed in conjunction with a senior university librarian, pilot tested on PubMed and individualised for each database (Appendix S1). Key words included in the search were: (“near miss” or “neonatal illness severity score” or “neonatal disease severity score” or “neonatal morbidity” or “near miss, healthcare”) AND (neonatal or perinatal or “perinatal care” or newborn or new-born or “infant, newborn”) AND (audit or “clinical audit” or review) NOT “systematic review”.

Electronic databases MEDLINE (PubMed), Embase (Elsevier), Scopus (Elsevier), CINAHL (EBSCO), LILACS (BIREME – PAHO – WHO website) and SciELO (Web of Science) were searched from the databases’ inception to 4th February 2023 with no publication date or language limits imposed.

### Inclusion and exclusion criteria

#### Participants

The participants are pregnant people and/or babies which the neonatal near miss audits have included. There were no further requirements (e.g. gravidity, age, or health of baby or pregnant person, etc.) on the participants that were involved in the audits. Audits were only included if they were conducted in hospitals and health services. Studies undertaken in high, middle, and low-income countries worldwide were considered for inclusion.

#### Intervention

NNM audit was defined as the process of identifying NNM cases, collecting and analysing the information with the involvement of a multidisciplinary team [8]. The inclusion of at least two different teams (e.g. obstetricians, paediatricians/ neonatologists, midwives, anaesthetists etc.) during the review process was considered adequate. We acknowledge that different health services might label their audit activities differently, thus, audits, clinical audits, reviews, and morbidity reviews were screened and included given they fulfilled the above description of a “multidisciplinary NNM audit”. There are no standard, internationally agreed criteria for NNM [10, 17], thus any article using a NNM definition pre-specified by the study’s authors was included.

#### Comparators

The use of audits was compared against standard practice and/or baseline periods with the absence of audits in the studied facilities.

### Outcome measures

#### Primary outcomes

- Perinatal mortality rates.
- Perinatal morbidity rates.
- NNM rates.

#### Secondary outcomes

- Identification of contributing care factors for neonatal near misses.
- Quality of care in participating facilities (measured using a method, tool or scale specified by the study authors).

### Additional context information

Due to the current review being foundational work within this field, additional data including feasibility, cost-effectiveness, ability to generate targeted recommendations, perceived facilitators and barriers, and the experience of staff and parents involved were prespecified and extracted from multidisciplinary NNM audits.

#### Types of sources included

Both randomized and observational studies were included (randomized controlled trials, cluster-randomized trials, quasi-randomized controlled trials, controlled before-and-after, interrupted time-series, case-control, cohort studies, cross sectional studies and case series). Peer-reviewed qualitative or mixed methods articles were included only to gain understanding on the additional context information as described above.

Conference abstracts, letters, studies duplicating validation data from previous studies, grey literature and unpublished studies were excluded.

#### Study selection

Identified references were imported into COVIDENCE / 2019 [29] and duplicates were removed. Following a pilot test, screening of title and abstracts and of articles selected for full-text review was performed independently by two researchers (“Author 1” and “Author 2”). Conflicts were resolved through discussion or by consultation with a third senior researcher (“Author 3”).

#### Extraction of information, evaluation of methodological quality and synthesis of results

Two independent reviewers (“Author 1” and “Author 2”) performed data extraction (using a customised template) and assessed methodological quality using JBI instruments relevant to each study design [30, 31]. Disagreements were resolved by consensus or with a third reviewer (“Author 3”). Meta-analysis was planned using Stata 14 software (Stata Corp LLC, Texas, USA). A sensitivity analysis to evaluate the stability of the results and a subgroup analysis by income of country, or rural/urban setting were proposed. A narrative summary of data was planned where meta-analysis of outcomes data was not possible. The GRADE [32] approach was planned for assessing certainty of evidence. Narrative summary was also planned for description of cost effectiveness, ability to generate targeted recommendations, perceived facilitators and barriers.

To our knowledge, there is no core outcome set available or applicable to this review. A parent with lived experience (DP) was involved in the development and conduct of the study as one of the investigators of this systematic review.

#### Deviations to the protocol

One change from the published protocol [28] was made before completion of data extraction. The change was to include “contributing care factors” among the secondary outcomes, and screening criteria were altered accordingly.

## Results

### Search results

**Fig. 1:**
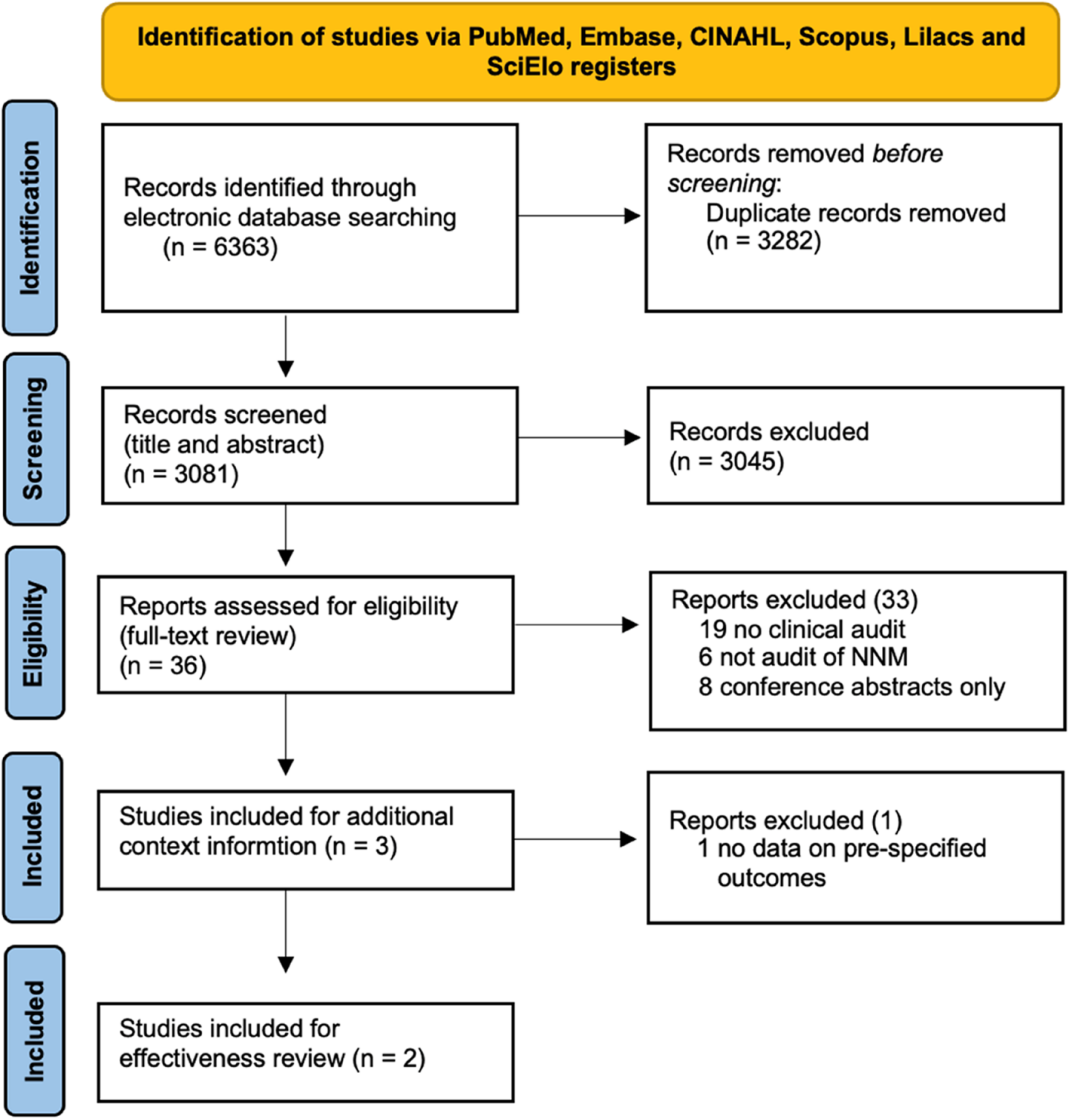
Search results and study selection and inclusion process

A total of 3081 unique records (after 3282 duplicates were removed from 6363 retrieved) underwent title and abstract screening, of which 36 studies were identified for full-text review (Table S1). Three multidisciplinary NNM audits [18, 33, 34] were identified for context information. Two of them [18, 33] were included for the effectiveness review (12,234 total births, 149 NNM, 41 stillbirths and 18 neonatal deaths) (Fig. 1 and Table 1).

**Table 1:** Characteristics of multidisciplinary NNM audits

Author	Year	Country/ Setting	Design	Sample size (births)	NNM n (n/1000LB)	Stillbirth n (n/1000LB)	Neonatal death n (n/1000LB)	Audit period	Type of audit / intervention	NNM / stillbirth / neonatal death post audit
DeKnif et al*. * [33]^d^	2010	South Africa, 1 regional hospital	Cohort (no baseline control period described)	10,117	125 (12.3)	14^a^ (1.38)	15 (1.48)	2008- 2009 (24 months)	Case file review and confidential enquiry to identify avoidable factors, missed opportunities and substandard care. Obstetrics / gynaecology and paediatricians meeting weekly, one author reviewed for avoidable factors Problems classified in 3 groups: patient associated, administrative, medical and personnel associated problems	No data
Bonnaerens et al*.* [18]^d^	2011	Belgium, 1 tertiary hospital	Cohort (no baseline control period described)	2117	24 (11.4)	12^b^ (5.2)	3 (1.4)	2010 (12 months)	Case notes reviewed by obstetricians and neonatologists Problems classified in 5 categories: fetal monitoring error, labour management error, instrumental vaginal delivery for fetal distress within 2 h of second stage, non-obstetric medical complications, preterm births or accidental and unavoidable cases at term	No data
Rana et al*.* [34]^e^	2018	Nepal, 1 district hospital and 14 community birthing centres	Modified time-series	1386	30 (22)	15^c^ (10.8)	11 (7.9)	2015 (12 months)	Monthly multidisciplinary meetings with case notes reviewed by consultant obstetricians and paediatricians This was part of a modified time series evaluation incorporating assessment and dissemination before, throughout and after the intervention	No data

Author	Identified local perinatal risk factors	Targeted recommendations post audit	Change in quality of care post audit	Experience of staff involved	Experience of parents involved	Cost- effect	Facilitators and/or barriers
DeKnif et al*.* [33]^d^	Yes. Incorrect management of second stage of labour. Patient refusal of medical treatment. No detection / no reaction to fetal distress. Inadequate facilities	Yes. Further training in management of labour. introduction of a checklist to promote decision making in the labour ward. Protocols for pain relief in labour in combination with counselling at the antenatal clinic about what to expect in labour	No data	No data	No data	No data	No data
Bonnaerens et al*.* [18]^d^	Yes. Violation of fetal monitoring and management of labour guidelines. Inadequate active management of labour	Yes. Adaptation of local protocol for management of labour including focus on discussing harms and benefits with the parents	No data	No data	No data	No data	No data
Rana et al*.* [34]^e^	Yes. Increased numbers of NNM were due to birth asphyxia, very low birthweight, neonatal sepsis	Yes. Increased availability and readiness of medicines/ equipment in the hospital. Increased awareness in the community to reduce risk factors, including home births and early age pregnancy	No data	Yes. Well received once it is based on a successful outcome, not a death. Useful to discuss clinical and managerial aspects of neonatal near-miss case management. (Measured by interviews and focus groups)	No data	US$ 800 per birthing centre	Barriers: time constraints, financial support, low adherence to audit guidelines

*NNM* Neonatal near-miss, *LB* Live births

^a^Intrapartum stillbirths only

^b^Intra-uterine and intrapartum stillbirths

^c^Unclear stillbirth definition

^d^Included for effectiveness review

^e^Included for additional context information only

### Study characteristics and overview

Three studies were identified as multidisciplinary NNM audits and analysed for context information [18, 33, 34]. Two were prospective cohort studies [18, 33] and one was a mixed-methods modified time-series [34]. One study was performed in a high-income country (Belgium) [18], while the other two were from upper (South Africa) [33] and lower (Nepal) [34] middle-income countries (classified using World Bank Country classifications [24]). The level of healthcare facilities analysed varied from a tertiary hospital (Belgium), a secondary hospital (South Africa) and a district hospital including community birthing centres (Nepal) with a length of audit intervention from 12 to 24 months.

The a priori NNM definitions used varied extensively, from established metabolic acidosis at birth to consideration of clinical observations, interventions and judgements about organ dysfunction and laboratory tests (Table 2). The NNM rate ranged from 11.4 [18] to 22 [34] per 1000 live births, while the NNM to mortality ratio ranged from 1.15 [34] to 4.3 [33]. The severe neonatal outcome ratio (including neonatal deaths and neonatal near miss cases) [17] ranged from 12.8 [18] to 29.9 [34] per 1000 live births.

**Table 2:** Neonatal near miss definitions

**Author**	**NNM definition**
DeKnif et al*.* [33]	**NNM criteria described by Avenant:** Respiratory failure/dysfunction: need for intubation and ventilation including nasal CPAP Cardiac failure/dysfunction: need for adrenalin, other inotropic support or volume expansion CNS failure/dysfunction: any convulsions or need for therapeutic anticonvulsants Hypovolaemia: need for blood transfusion or volume expansion Haematological failure/dysfunction: need for phototherapy or exchange blood transfusion, need for neupogen to increase white cells Endocrine failure/dysfunction: need to treat hypoglycaemia (additional glucose) Renal failure/ dysfunction: haematuria and/or oliguria / anuria Immunologic failure/ dysfunction (congenital infection): CRP greater than or equal to 10 or raising CRP Muscle-skeletal morbidity: any fracture GIT/Hepatic failure/dysfunction
Bonnaerens et al*.* [18]	**Established metabolic acidosis at birth:** Arterial pH < 7.05 or venous pH < 7.17, in association with base excess ≤ -10 mmol/L In cases of sampling or analysis-error, neonates with persistently low Apgar score of ≤ 6 after 5 min were considered clinically at risk for metabolic acidosis
Rana et al*.* [34]	**Adapted from WHO Multi-country Survey (Pileggi-Castro):** Clinical sign-based criteria: Loss of consciousness > five minutes, persistent bradycardia (heart rate < 80 beats/minute), persistent tachycardia (heart rate > 200 beats/minute), poor capillary refill (> three seconds), acute central cyanosis in room air, gasping respiration, anuria lasting > six hours, visible haematuria, failure to form clots (bedside clotting time > seven minutes, clots that break easily, the absence of clotting from iv sites or suture lines after 7–10 min), use of therapeutic intra venous antibiotics, visible jaundice in first 24 h, uncontrollable fit/status epilepticus Danger signs-based criteria: not breathing even after stimulation and suction, fast breathing/pneumonia, severe chest indrawing, lethargy/unconsciousness, poor sucking/unable to suck mother’s milk in term baby, hypothermia (temperature < 36.5°C), fever (> 100.4°F/ > 38°C) reddish umbilicus extending to the surrounding skin/single boil/ 10 pustules on the skin, convulsion, nasal flaring, bulging anterior fontanelle, grunting, jaundice within 24 h of birth (visible on palms and soles), diarrhoea with severe dehydration, persistent vomiting; conditions at birth-based criteria very low birthweight (< 1500 g), preterm (< 30 weeks)

The studies described clinical audits with involvement of multidisciplinary teams (at least two teams e.g., obstetricians and paediatricians or neonatologists participating in case reviews). Two studies [18, 33] reported pre-specified classification criteria for substandard care. One study [34] included data collected through in-depth interviews, focus groups and observation checklists with policymakers, district-level managers, healthcare workers and mothers at baseline and end of the intervention. The characteristics of the studies are described in Table 1.

### Outcomes

#### Primary outcomes

No study compared rates of perinatal mortality, perinatal morbidity and/ or NNM rates before and after an audit intervention.

#### Secondary outcomes

##### Identification of contributing care factors for NNM

Two of the identified multidisciplinary NNM audits with pre-specified classification criteria for contributing care factors of NNM reported the rate and distribution of contributing factors. DeKnijf et al. [33] from South Africa, classified contributing care factors in three groups (patient associated, administrative, medical personnel associated problems). These factors were present in 37% of NNM cases and the major problem identified was incorrect management of second stage of labour. Bonnaerens et al. [18] from Belgium, classified in five different groups (fetal monitoring error, labour management error, instrumental vaginal delivery for fetal distress within two hours of second stage, non-obstetric medical complications, preterm births or accidental and unavoidable cases at term). They found contributing care factors in 46% of the audited NNM cases and identified instrumental vaginal delivery for fetal distress within two hours of second stage as their main local avoidable factor.

##### Change in quality of care in participating facilities

No study provided quantitative data on changes in quality of care in participating facilities.

### Additional context information

#### NNM generating targeted recommendations

Two of the identified multidisciplinary NNM audits [18, 33] reported the rate and distribution of contributing factors and were able to generate local targeted recommendations aiming to improve quality of care (e.g. updating of guidelines, focused upskilling of personnel).

#### NNM audits and their cost-effectiveness

Rana et al. reported a cost of US$ 800 per birthing centre for the implementation of the audit program [34]. No formal analysis of cost-effectiveness was available.

#### Experience of staff and parents involved in NNM audits

One multidisciplinary NNM audit was performed in 15 rural health facilities in Nepal and described positive feedback from staff, with audits perceived as a useful platform to discuss clinical and managerial aspects of maternal and neonatal near-miss case management [34]. This systematic review did not find any study reporting parent engagement in NNM audits.

#### NNM audits and their perceived facilitators and barriers

Rana et al. reported barriers to NNM which included time constraints, lack of financial support, low adherence to audit guidelines, lack of protected time and difficulties with setting the near-miss criteria for different level of health facilities [34].

#### NNM audits and quality of care

This review found no quantitative data regarding change in quality of care, however, one study provided a qualitative description of improvement in quality of care. Rana et al. [34] performed 14 in-depth interviews and six focus group discussions (including 13 healthcare workers, 28 healthcare volunteers and 24 mothers) before the NNM audit intervention and 21 in-depth interviews and five focus groups (with 14 healthcare workers, 23 healthcare volunteers and 15 mothers) at the end of the audit intervention. The results included a perceived increase in staff confidence for managing near-miss cases and perceived improvement in healthcare workers’ knowledge and clinical skills. Additionally, an improvement was shown on the availability of resources for quality care, including life-saving medicines and equipment at health facilities.

### Methodological quality of studies, and certainty of evidence

The methodological quality scores for studies included in the effectiveness review are provided in Table S2. The cohort study scores were 82% [33] and 90% [18] of the total possible score. Neither study reported long-term follow up and DeKnif et al. [33] did not report strategies to address incomplete follow up.

For primary outcomes, GRADE analysis could not be conducted because of the absence of numerical data. Applying GRADE criteria [32] to the secondary outcome reported (identification of contributing factors for NNM) showed a very low certainty of evidence (downgraded due to imprecision) (Table S3). GRADE analysis was not conducted on qualitative data or additional context information.

## Discussion

This systematic review of NNM audits identified two studies reporting on identification of contributing care factors but no eligible studies reporting on changes in perinatal mortality, morbidity and NNM rates post intervention. Three multidisciplinary NNM audits were identified and had narrative synthesis for additional context information. Although data is scarce, NNM audit appears to be feasible, able to generate targeted recommendations and improve perceived knowledge and confidence of healthcare workers. This review highlights the large void of academic literature on in-depth NNM audits and launches a Call to Action for further studies to evaluate the effectiveness of NNM audits comprising standardised NNM definitions and to define best practice in this area.

NNM audits, and the better-established perinatal death audits, have been regarded as instruments to reduce perinatal adverse outcomes. Nevertheless, there is a scarcity of data on effectiveness of both types of audits. A 2020 Cochrane review on impact of death audits in reducing maternal, perinatal and child mortality found improvement in quality of care in a high-income setting but highlighted the need for further operational research of audits especially in low- and middle-income countries [35]. Although there were no data available on effectiveness of NNM audits for patient-centred outcomes (change in mortality, morbidity and NNM), NNM audits were able to identify, rate and classify contributing care factors. The proportion of contributing factors described by the two studies included in this review ranged from 37% in South Africa [33] to 46% in Belgium [18]. Likewise, perinatal mortality audits from the Netherlands [36], United Kingdom [37], New Zealand [38] and Australia [6] have revealed substandard care factors in up to 60% of intrapartum stillbirths. To improve patient’s care through a clinical audit, recognition of contributing factors and generation of targeted recommendations are key intermediate steps. The findings of these two studies demonstrate that NNM audits can accomplish a critical first step on the pathway to improvement in perinatal outcomes. The paucity of reports of any aspect of effectiveness of NNM audit eligible for inclusion in the review is concerning. While trials randomised at patient level to test our study question would be difficult or unethical for both NNM and perinatal death audits, other strategies such as large, well-designed before-and-after studies within services or comparisons between services could contribute valuable evidence.

Perinatal audits may also have value in helping parents to understand what happened to their babies and assist both parents and their healthcare providers in care and decision-making in subsequent pregnancies [39]. Qualitative research from low-income countries exploring mother’s perceptions on their lived experiences of NNM has highlighted the need for improvement in communication between health providers and families, and better access to good quality healthcare services [40, 41]. Although studies show that parent engagement in perinatal mortality audit improves the review process [42, 43] the same has not been done for NNM.

Perinatal mortality and NNM audits appear to have similar barriers to their implementation. These include the lack of managerial support, lack of protected time, difficulties with engagement and training of multidisciplinary teams, fear of blaming and litigation [7, 34, 44]. Additionally, a survey from high-income country perinatal heath care workers disclosed the lack of NNM consensus definition, lack of robust discussions, and difficulty in implementing improvement strategies as important barriers to NNM audits [45]. Nevertheless, very limited data suggests that NNM audits are perceived as in-depth analysis of successful cases from which lessons can be learned [34, 46]. This positive view of NNM audit could be seen as another advantage of a combined perinatal mortality and NNM audit, breaking down the fear of such activity. However, well-designed studies are needed to access the results from this joint approach.

The variation in NNM definitions is recognized as an important barrier to comparison, benchmarking, and generalization of NNM audit results [10, 17, 47]. A recent literature review found seven different NNM definitions and highlighted the need for consensus [48]. Given these findings, unsurprisingly, the identified NNM audits in this systematic review used varying NNM criteria.

The current review supports a Call to Action for NNM audits (Table 3). There is a need for services to both innovate in developing NNM audits because there is no existing international consensus or template. It is critical that services report their methods and outcomes to enable the development of such a consensus. Use of a formal audit methodology, clear NNM definitions and evidence-based strategies for implementation of recommendations are paramount for effective NNM audit programs [8]. There is need for well-designed NNM audit research to confirm their effectiveness. Although not free of confounders, large before-and-after studies within services or comparisons between services could contribute evidence. Future studies should include evaluation of parents’ engagement in such audits.

**Table 3:** Call to Action for NNM audits

**NNM audits can uncover factors contributing to perinatal mortality and morbidity, which is a key step to reduce perinatal adverse outcomes** **This review has identified important gaps in the area and launches a Call to Action:**		
**Gaps Identified**		**Call to Action**
**1.**	No study reported on effectiveness of NNM audits	There is need for future better and well-designed NNM audit research to confirm effectiveness of NNM audits
**2.**	Paucity of multidisciplinary NNM audits using a formal audit methodology	There is need for adoption of a formal audit methodology and inclusion of a multidisciplinary team when conducting NNM audits
**3.**	Wide variation in NNM definitions used for NNM audits	Consensus NNM definitions are needed to allow comparison, benchmarking, and generalization of NNM audit results
**4.**	No data available regarding parents’ engagement in NNM audits	Future research should evaluate parents’ engagement in NNM audits to help delineating the best practice

### Strengths and limitations

The strengths of this review include the rigorous, pre-defined methods [28], the comprehensive search strategy, and the use of a broad range of bibliographical databases to maximize inclusion of possible relevant studies.

The outcomes of this review were limited by the paucity of data and well-designed studies including measurement of important patient-centred outcomes. No formal analysis of the likelihood of publication bias was not possible, but importantly, we could not determine whether the scarcity of published reports on NNM audit is because the process itself is uncommon, because its effectiveness has rarely been audited, or because papers describing outcomes have not been written or published.

## Conclusions

This systematic review provides a summary of the existing evidence on NNM clinical audits. NNM audits can help with identifying contributing care factors which is an essential intermediate step for improvement of care. Nevertheless, there is a lack of evidence for the effectiveness of NNM audits on key outcomes such as perinatal mortality, morbidity and NNM. This review supports a Call to Action for future NNM audits. Higher quality studies measuring important clinical outcomes are needed. These audits should use definitions that have been achieved by consensus so that in the future, benchmarking can be facilitated, and studies can be compared and meta-analysed.

### Supplementary Information

**Additional file 1: Appendix S1.** Search strategy.

**Additional file 2: Table S1.** Excluded studies.

**Additional file 3: Table S2.** Critical appraisal of included cohort studies.

**Additional file 4: Table S3.** Summary of Findings.

## Data Availability

The datasets used and/or analysed during the current study available from the corresponding author on reasonable request.

## Abbreviation

NNM: Neonatal near-miss
