# Supplementary material for: Neonatal near-miss audits: a systematic review and a call to action
Source: BMC Pediatr. 2023 Nov 17;23:573. doi: 10.1186/s12887-023-04383-6 (PMC10655277; doi:10.1186/s12887-023-04383-6)
Supplement: Supplementary file 1 — Additional file 1: Appendix S1. Search strategy. [file 12887_2023_4383_MOESM1_ESM.docx]

**Appendix S1: Search strategy**

| **Search** | **Query** | **Records retrieved** |
| --- | --- | --- |
| **PubMed** | ("near miss*"[tiab] OR "neonatal illness severity score*"[tiab] OR "neonatal disease severity score*"[tiab] OR "neonatal morbidity"[tiab] OR "Near Miss, Healthcare"[Mesh])  AND (neonat*[tiab] OR perinatal[tiab] OR "Perinatal Care"[Mesh] OR newborn*[tiab] OR new-born*[tiab] OR "Infant, Newborn"[Mesh])  AND (audit*[tiab] OR "Clinical Audit"[MeSH] OR review*[tiab])  NOT “systematic review” [ti] | 1,238 |
| **Embase** | ("near miss*":ti,ab OR "neonatal illness severity score*":ti,ab OR "neonatal disease severity score*":ti,ab OR "neonatal morbidity":ti,ab OR 'near miss, (health care)'/exp)  AND (neonat*:ti,ab OR perinatal:ti,ab OR 'perinatal care'/exp OR newborn*:ti,ab OR new-born*:ti,ab OR 'newborn'/exp)  AND (audit*:ti,ab OR 'clinical audit'/exp OR review*:ti,ab)  NOT "systematic review":ti | 1755 |
| **Scopus** | (TITLE-ABS("near miss*") OR TITLE-ABS("neonatal illness severity score*") OR TITLE-ABS("neonatal disease severity score*") OR TITLE-ABS("neonatal morbidity"))  AND (TITLE-ABS("neonat*") OR TITLE-ABS("perinatal") OR TITLE-ABS("newborn*") OR TITLE-ABS("new-born*"))  AND (TITLE-ABS("audit*") OR TITLE-ABS("review*"))  AND NOT TITLE("systematic review") | 1398 |
| **CINAHL** | ((TI "near miss*" OR AB "near miss*") OR (TI "neonatal illness severity score*" OR AB "neonatal illness severity score*") OR (TI "neonatal disease severity score*" OR AB "neonatal disease severity score*") OR (TI "neonatal morbidity" OR AB "neonatal morbidity")  AND ((TI neonat* OR AB neonat*) OR (TI perinatal OR AB perinatal) OR (MH "Perinatal Care+") OR (TI newborn* OR AB newborn*) OR (TI new-born* OR AB new-born*) OR (MH "Infant, Newborn+"))  AND ((TI audit* OR AB audit*) OR (MH "Audit+") OR (TI review* OR AB review*))  NOT TI "systematic review" | 1922 |
| **LILACS** | ("near miss" OR "near misses" OR "neonatal illness severity score" OR "neonatal illness severity scores" OR "neonatal disease severity score" OR "neonatal disease severity scores" OR "neonatal morbidity" OR MH:"Near Miss, Healthcare")  AND (neonat* OR perinatal OR MH:"Perinatal care" OR newborn* OR new-born* OR MH:"Infant, Newborn")  AND (audit OR audits OR audited OR auditing OR MH:"Clinical Audit" OR review OR reviews OR reviewed)  AND NOT TI:"systematic review" | 47 |
| **SciELO** | ("near miss" “near misses” OR "neonatal illness severity score" OR "neonatal disease severity score" OR "neonatal morbidity")  AND (neonatal OR perinatal OR "perinatal care" OR newborn OR newborns OR new-born OR)  AND (audit OR audits OR "clinical audit" OR review OR reviews) | 3 |
| No limits imposed | | |
| Search conducted on to 4th February 2023 | | |
