# Supplementary material for: Neonatal near-miss audits: a systematic review and a call to action
Source: BMC Pediatr. 2023 Nov 17;23:573. doi: 10.1186/s12887-023-04383-6 (PMC10655277; doi:10.1186/s12887-023-04383-6)
Supplement: Supplementary file 2 — Additional file 2: Table S1. Excluded studies. [file 12887_2023_4383_MOESM2_ESM.docx]

| **Table S1:** Excluded studies | | |
| --- | --- | --- |
| **Number** | **Reference** | **Summary comments for exclusion** |
| **1** | Bakari A, Bell AJ, Oppong SA, Bockarie Y, Wobil P, Plange-Rhule G, et al. Neonatal near-misses in Ghana: a prospective, observational, multi-center study. BMC Pediatr. 2019;19(1):509-. | No clinical audit activity |
| **2** | Benjamin R, Clement-Jones M. Audit on unexpected admission of term babies to SCBU-1 year data. BJOG: An International Journal of Obstetrics and Gynaecology. 2013;120:30. | Conference abstract only |
| **3** | Day LT, Mussell F, Khatun H, Verbiest R, Biswas L, Folia R, et al. Sustaining perinatal audit in the high stillbirth rate setting - A 20 year journey in rural Bangladesh. BMC Pregnancy and Childbirth. 2017;17. | Conference abstract only |
| **4** | de Lima THB, Katz L, Kassar SB, Amorim MM. Neonatal near miss determinants at a maternity hospital for high-risk pregnancy in Northeastern Brazil: a prospective study. BMC Pregnancy Childbirth. 2018;18(1):401-8. | No clinical audit activity |
| **5** | DeMorais LR, Patz BC, Campanharo FF, Dualib PM, Cecatti JG, Sun SY, et al. Maternal and neonatal near miss of patients with type 1 diabetes: Preliminary results. International Journal of Gynecology and Obstetrics. 2018;143:587-8. | Conference abstract only |
| **6** | Dickins D, Neville A, Clement-Jones M, Benjamin R, Whitham L. Audit of unexpected term admissions to SCBU at liverpool women's Hospital. Archives of Disease in Childhood: Fetal and Neonatal Edition. 2014;99:A115. | Conference abstract only |
| **7** | Drife JO. Perinatal audit in low- and high-income countries. Semin Fetal Neonatal Med. 2006;11(1):29-36. | No clinical audit activity |
| **8** | Enweronu-Laryea CC, Onwona-Agyeman K, Ayibor PK, Annan FB, Gyakari EO, Vanotoo LA. Retrospective review of neonatal morbidity and mortality at public referral hospitals in Greater Accra Region of Ghana:2013-2014. J Neonatal Perinatal Med. 2020;13(1):105-13. | No clear NNM |
| **9** | Geerlings E, Kaselitz E, Aborigo RA, Williams J, Youngblood J, Avrakotos A, et al. 'I am still confused as to what caused the problem': Perceptions of mothers on communication regarding newborn illness and death in Northern Ghana. Global Public Health. 2019;14(12):1784-92. | No clinical audit activity |
| **10** | Guimarães Maia MR, Aparecida Pimenta Ferrari R, Aparecida Maciel Cardelli A, Harumi Higarashi I, de Barros Carvalho MD, Pelloso SM. Neonatal near miss in the intensive care unit. Revista Brasileira de Enfermagem. 2020;73:1-6. | No clinical audit activity |
| **11** | Helps Ä, Leitao S, O'Donoghue K. The way forward in perinatal mortality reviews. BJOG: An International Journal of Obstetrics and Gynaecology. 2019;126:73. | Conference abstract only |
| **12** | Ignatko IV, Rodionova AM, Kardanova MA, Bogomazova IM, Samusevich AN, Florova VS, et al. Neonatal near miss - «almost dead» newborns: Criteria of evaluation and value in perinatalaudit. Voprosy Ginekologii, Akusherstva i Perinatologii. 2017;16(2):40-8. | No clinical audit activity |
| **13** | Johanson RB, Rigby C. The "ASQUAM" programme: Improving the standards of maternity care, using democratic prioritisation, guidelines and audit. British Journal of Clinical Governance. 2002;7(2):112-21. | No clinical audit activity |
| **14** | Kaselitz E, James KH, Aborigo RA, Agorinya I, Moyer CA, Williams J. Understanding the gap in emergency obstetric and neonatal care in Ghana through the PREventing Maternal And Neonatal Deaths (PREMAND) study. International Journal of Gynecology & Obstetrics. 2019;145(3):343-9. | No clinical audit activity |
| **15** | Kerkhofs C, De Bruyn C, Mesens T, Theyskens C, Vanhoestenberghe M, Bruneel E, et al. Identification of peripartum near-miss for perinatal audit. Facts Views Vis Obgyn. 2014;6(4):177-83. | No clinical audit activity |
| **16** | Klingenberg C, Olomi R, Oneko M, Sam N, Langeland N. Neonatal morbidity and mortality in a Tanzanian tertiary care referral hospital. Ann Trop Paediatr. 2003;23(4):293-9. | No clear NNM |
| **17** | Manandhar SR, Manandhar DS, Adhikari D, Shrestha JR, Rai C, Rana H, et al. Neonatal near miss cases of different health facilities. Journal of Nepal Paediatric Society. 2014;34(2):115-8. | No clinical audit activity |
| **18** | Mersha A, Bante A, Shibiru S. Factors associated with neonatal near-miss in selected hospitals of Gamo and Gofa zones, southern Ethiopia: nested case-control study. BMC Pregnancy & Childbirth. 2019;19(1):1-8. | No clinical audit activity |
| **19** | Mikhailov A. Perinatal audit of intranatal death and neonatal near-miss. Journal of Perinatal Medicine. 2017;45:179. | Conference abstract only |
| **20** | Miltenburg AS, van Elburg RM, Kostense PJ, van Geijn HP, Bolte AC. Neonatal morbidity in term neonates is related to gestational age at birth and level of care. J Perinat Med. 2011;39(5):605-10. | No clear NNM |
| **21** | Morais LR, Patz BC, Campanharo FF, Dualib PM, Sun SY, Mattar R. Neonatal Near Miss among Newborns of Women with Type 1 Diabetes Mellitus. Obstetrics and Gynecology International. 2019;2019:8594158. | No clinical audit activity |
| **22** | Moyer CA, Aborigo RA, Kaselitz EB, Gupta ML, Oduro A, Williams J. PREventing Maternal And Neonatal Deaths (PREMAND): a study protocol for examining social and cultural factors contributing to infant and maternal deaths and near-misses in rural northern Ghana. Reprod Health. 2016;13:20. | No clinical audit activity |
| **23** | Moyer C, Aborigo R, Kaselitz E, James K, Chatio S, Williams J. Preventing maternal and neonatal deaths in rural northern ghana (premand): Using social autopsy and gis to understand neonatal deaths and near-misses. Annals of Global Health. 2017;83(1):196. | Conference abstract only |
| **24** | Mukhtar-Yola M, Iliyasu Z. A review of neonatal morbidity and mortality in Aminu Kano Teaching Hospital, northern Nigeria. Trop Doct. 2007;37(3):130-2 | No clear NNM |
| **25** | Musooko M, Kakaire O, Nakimuli A, Nakubulwa S, Nankunda J, Osinde MO, et al. Incidence and risk factors for early neonatal mortality in newborns with severe perinatal morbidity in Uganda. Int J Gynaecol Obstet. 2014;127(2):201-5. | No clinical audit activity |
| **26** | Raheem M, Orefice R. An audit of umbilical artery cord ph <7. Journal of Paediatrics and Child Health. 2020;56(SUPPL 1):113. | Conference abstract only |
| **27** | Rana HB, Banjara MR, Joshi MP, Kurth AE, Castillo TP. Assessing maternal and neonatal near-miss reviews in rural Nepal: an implementation research study to inform scale-up. Acta Paediatr. 2018;107(S471):17-23. | No data on primary or secondary outcomes |
| **28** | Ronsmans C, Cresswell JA, Goufodji S, Agbla S, Ganaba R, Assarag B, et al. Characteristics of neonatal near miss in hospitals in Benin, Burkina Faso and Morocco in 2012-2013. Tropical Medicine & International Health. 2016;21(4):535-45. | No clinical audit activity |
| **29** | Tekelab T, Chojenta C, Smith R, Loxton D. Incidence and determinants of neonatal near miss in south Ethiopia: a prospective cohort study. BMC Pregnancy Childbirth. 2020;20(1):354. | No clinical audit activity |
| **30** | Tekola AF, Baye G, Amaje E, Tefera K. Neonatal near misses and associated factors among mother's who give a live neonate at Hawassa City governmental hospitals, 2019: a facility based cross-sectional study design. BMC Pregnancy & Childbirth. 2021;20(1):1-9. | No clinical audit activity |
| **31** | Todd S, Bowen J, Ibiebele I, Patterson J, Torvaldsen S, Ford J, et al. A composite neonatal adverse outcome indicator using population-based data: An update. International Journal of Population Data Science. 2020;5(1). | No clinical audit activity |
| **32** | Tosif S, Jatobatu A, Maepioh A, Subhi R, Francis KL, Duke T. Cause-specific neonatal morbidity and mortality in the Solomon Islands: An assessment of data from four hospitals over a three-year period. J Paediatr Child Health. 2020;56(4):607-14. | No clear NNM |
| **33** | Udo JJ, Anah MU, Ochigbo SO, Etuk IS, Ekanem AD. Neonatal morbidity and mortality in Calabar, Nigeria: a hospital-based study. Niger J Clin Pract. 2008;11(3):285-9. | No clear NNM |
| **34** | Wick L. Survival and negotiation: narratives of severe (near-miss) neonatal complications of Syrian women in Lebanon. Reproductive Health Matters. 2017;25(sup1):27-34. | No clinical audit activity |
