# Supplementary material for: Neonatal near-miss audits: a systematic review and a call to action
Source: BMC Pediatr. 2023 Nov 17;23:573. doi: 10.1186/s12887-023-04383-6 (PMC10655277; doi:10.1186/s12887-023-04383-6)
Supplement: Supplementary file 3 — Additional file 3: Table S2. Critical appraisal of included cohort studies. [file 12887_2023_4383_MOESM3_ESM.docx]

| **Table S2. Critical appraisal of included cohort studies.**  JBI Critical Appraisal Checklist for Cohort Studies (31). | | | | | | | | | | | | |
| --- | --- | --- | --- | --- | --- | --- | --- | --- | --- | --- | --- | --- |
| **Study** | **Q1** | **Q2** | **Q3** | **Q4** | **Q5** | **Q6** | **Q7** | **Q8** | **Q9** | **Q10** | **Q11** | **Score** |
| DeKnif *et al.* (33) | Y | NA | Y | Y | Y | Y | Y | N | Y | N | Y | 9/11 |
| Bonnaerens *et al.* (18) | Y | NA | Y | Y | Y | Y | Y | N | Y | N/A | Y | 10/11 |
| Y, yes; N, no; NA, not applicable Q1: Were the two groups similar and recruited from the same population?  Q2: Were the exposures measured similarly to assign people to both exposed and unexposed groups?  Q3: Was the exposure measured in a valid and reliable way?  Q4: Were confounding factors identified?  Q5: Were strategies to deal with confounding factors stated?  Q6: Were the groups/participants free of the outcome at the start of the study (or at the moment of exposure)?  Q7: Were the outcomes measured in a valid and reliable way?  Q8: Was the follow up time reported and sufficient to be long enough for outcomes to occur?  Q9: Was follow up complete, and if not, were the reasons to loss to follow up described and explored?  Q10: Were strategies to address incomplete follow up utilized?  Q11: Was appropriate statistical analysis used? | | | | | | | | | | | | |
