# Supplementary material for: Neonatal near-miss audits: a systematic review and a call to action
Source: BMC Pediatr. 2023 Nov 17;23:573. doi: 10.1186/s12887-023-04383-6 (PMC10655277; doi:10.1186/s12887-023-04383-6)
Supplement: Supplementary file 4 — Additional file 4: Table S3. Summary of Findings. [file 12887_2023_4383_MOESM4_ESM.docx]

| **Table S3: Summary of Findings** | | | | | |
| --- | --- | --- | --- | --- | --- |
| **NNM audits compared to standard practice and / or baseline period with absence of audits for reducing perinatal adverse outcomes** | | | | | |
| **Patient or population**: reducing perinatal adverse outcomes.  **Setting**: Healthcare facilities  **Intervention**: NNM audits  **Comparison**: standard practice and / or baseline period with absence of audits | | | | | |
| **Outcomes** | **Number of participants  (studies)** | **Certainty of the evidence (GRADE)** | **Relative effect (95% CI)** | **Anticipated absolute effects** | |
|  |  |  |  | **Risk with standard practice and / or baseline period with absence of audits** | **Risk difference with NNM audits** |
| **Table 3: Summary of Findings** | **Table 3: Summary of Findings** | **Table 3: Summary of Findings** | **Table 3: Summary of Findings** | **Table 3: Summary of Findings** | **Table 3: Summary of Findings** |
| Change in perinatal morbidity rates - not measured | - | **-** | - | - | - |
| Change in NNM rates - not measured | - | **-** | - | - | - |
| Identification of contributing care factors for NNM assessed with: rates of audits able to improve identification of local contributing care factors (18, 33) | 12234 (2 observational studies)  (18, 33) | ⨁◯◯◯ VERY LOW ^a^ | not pooled | not pooled | not pooled |
| Change in quality of care in participating facilities  assessed with: rates of studies describing improvement in healthcare facilities | - | **-** | - | - | - |

^a^ Downgraded due to risk of bias (not serious), inconsistency (not serious), indirectness (not serious) and imprecision (serious - different NNM definitions across studies)
